# Prevention and management of nosocomial infections in patients undergoing extracorporeal membrane oxygenation: a summary of best evidence

**DOI:** 10.3389/fmed.2026.1819134

**Published:** 2026-05-15

**Authors:** Dan-dan Xu, Shu-han Cai, Na-na Chen, Xin-bo Ding, Jing Ma, Chao Tian, Zhao-yang Li, Hui-lin Li, Fen Hu, Jin Li

**Affiliations:** 1 Department of Critical Care Medicine, Zhongnan Hospital of Wuhan University, Wuhan, China; 2 Clinical Research Center of Hubei Critical Care Medicine, Wuhan, China; 3 Health Science Center, Yangtze University, Jingzhou, China; 4 Department of Nursing, Zhongnan Hospital of Wuhan University, Wuhan, China

**Keywords:** critical illness, evidence summary, extracorporeal membrane oxygenation, infection prevention, nosocomial infection

## Abstract

**Purpose:**

This study aimed to systematically search, evaluate, and synthesize the latest evidence on the prevention and management of nosocomial infections in patients supported by extracorporeal membrane oxygenation.

**Methods:**

The study included clinical practice guidelines, expert consensus statements, systematic reviews, and other relevant publications. The quality of the included literature was rigorously assessed using appropriate tools such as AGREE II and AMSTAR. Data extraction and evidence synthesis were performed independently by two researchers. A comprehensive search was conducted across multiple databases and sources, including PubMed, Embase, the Cochrane Library, Web of Science, China National Knowledge Infrastructure, Wanfang Data, VIP, SinoMed, the National Guideline Clearinghouse, the Scottish Intercollegiate Guidelines Network, the Extracorporeal Life Support Organization website, and UpToDate. The search covered the period from the inception of each database up to October 31, 2025.

**Results:**

Fourteen studies met the inclusion criteria, comprising 2 clinical decisions, 4 guidelines, 4 expert consensus documents, 2 systematic reviews, and 2 cohort studies. All 14 studies were included following quality assessment. A total of 27 evidence items were synthesized and categorized into three domains: (1) infection prevention during extracorporeal membrane oxygenation initiation, (2) infection prevention during extracorporeal membrane oxygenation run, and (3) monitoring and treatment of extracorporeal membrane oxygenation-related infections.

**Conclusion:**

The prevention and control of nosocomial infections in patients receiving extracorporeal membrane oxygenation requires a comprehensive, bundle-based management strategy grounded in the best available evidence. The evidence synthesized in this study is both robust and practical. We recommend that clinical institutions adapt these evidence-based recommendations into specific clinical protocols and checklists tailored to their local context, with the ultimate goal of effectively reducing infection rates and improving clinical outcomes in patients receiving extracorporeal membrane oxygenation.

## 1 Introduction

The World Health Organization defines nosocomial infection (NI) as an infection that occurs during the process of care in a hospital or other healthcare facility, which was not present or incubating at the time of admission (1). NI is a common complication in patients receiving extracorporeal membrane oxygenation (ECMO) therapy, with a reported prevalence ranging from 8.8 to 64.0% and an incidence density of 1.7 to 85.4 per 1,000 ECMO-days (2). ECMO is a mode of extracorporeal life support that augments oxygenation, ventilation and/or cardiac output via cannulae connected to a circuit that pumps blood through an oxygenator and back into the patient (3). A recent systematic review indicated an observed NI incidence of 1,249 episodes per 1,000 ECMO-days, with ventilator-associated pneumonia and bloodstream infections being the most common types (4). Compared to non-infected patients, infected patients showed significantly lower ECMO survival and overall survival rates, with risk ratios of 0.84 (95% CI 0.74–0.96) and 0.80 (95% CI 0.71–0.90), respectively (4). Multiple meta-analyses (4, 5) have identified risk factors for NI in ECMO patients, including duration of mechanical ventilation, length of hospital stay, ECMO mode, immunosuppression, and heart transplantation. In addition, several ECMO-specific infection risks have not yet been fully elucidated. These include circuit biofilm formation, the complex interaction between anticoagulation and infection, and the adsorption of drugs by the circuit and oxygenator (6–8). It is the combination of these conventional and unique risk factors that poses substantial challenges to the prevention and control of NI in ECMO patients.

Current infection prevention measures for ECMO patients are often fragmented and inconsistently implemented across institutions, with significant variations in practice. Most existing protocols focus narrowly on care bundles for ventilator-associated pneumonia or catheter-related bloodstream infections, lacking a comprehensive, standardized, and systematic approach that covers the entire patient care pathway. Multiple studies have recommended five core measures for preventing NI in this population (9, 10): strict aseptic cannulation techniques, daily multidisciplinary assessment of circuit necessity, standardized oral care, goal-directed antibiotic stewardship, and bundled catheter care. A recent study evaluated a standardized prevention strategy incorporating chlorhexidine gluconate bathing and nasal decolonization (11). This multifaceted decontamination approach was associated with a significant reduction in NI risk (RR = 0.42 [95% CI, 0.23–0.60]) and multidrug-resistant organism acquisition (RR = 0.13 [0.03–0.56]), though no mortality difference was observed. For bloodstream infections, urinary tract infections, ventilator-associated pneumonia, and skin/soft tissue infections, Marcus et al. (12) emphasized the need for tailored, site-specific infection prevention and control protocols. However, it remains uncertain whether these conventional infection prevention bundles are sufficient for the ECMO population. A single-center study including 3,396 hospitalized patients and 288 ECMO patients revealed a higher incidence of NI in the ECMO group, despite receiving the same care and standardized protocols as non-ECMO patients (13). This suggests that routine infection control measures may be inadequate for this critically ill cohort, underscoring the need for specifically designed interventions tailored to the unique risks of ECMO support.

To address these challenges systematically, this study aims to synthesize the latest evidence related to the prevention and management of NI in ECMO patients, with the goal of providing a robust foundation for developing precise and effective prevention and control strategies in clinical practice.

## 2 Materials and methods

### 2.1 Study design and inclusion/exclusion criteria

To systematically identify, evaluate, and synthesize existing evidence for deriving clear and critical conclusions regarding infection management in ECMO patients, this study was registered with the Fudan University Center for Evidence-Based Nursing on October 9, 2025 (Registration No: ES20259062). The evidence-based question was clarified using the PIPOST framework from the Joanna Briggs Institute (JBI) Fudan University Center. The PIPOST elements were defined as follows: Population (P): adult ECMO patients; Intervention (I): measures related to ECMO infection prevention and management; Professionals (P): clinical healthcare providers; Outcomes (O): patient infection incidence; Setting (S): intensive care units; Type of evidence (T): clinical decisions, guidelines, systematic reviews, expert consensus documents, and relevant original studies.

Based on this framework, the inclusion criteria were established as follows: (1) target population: adult ECMO patients; (2) interventions: measures related to ECMO infection prevention and management; (3) professionals applying the evidence: clinical healthcare providers; (4) outcomes: patient infection incidence; (5) setting for evidence application: intensive care units; (6) types of evidence: clinical decisions, guidelines, systematic reviews, expert consensus documents, and relevant original studies. Exclusion criteria comprised: (1) documents with incomplete data, and (2) duplicate publications.

### 2.2 Search strategy

Guided by the 6S evidence model (14), a comprehensive literature search was conducted across multiple databases and official websites. These included the National Guideline Clearinghouse (USA), the Scottish Intercollegiate Guidelines Network, the Extracorporeal Life Support Organization (ELSO) website, UpToDate, PubMed, Embase, the Cochrane Library, Web of Science, China National Knowledge Infrastructure (CNKI), Wanfang Data, VIP, and SinoMed. The search period covered records from the inception of each database up to October 2025. Search terms encompassed the following key concepts: “extracorporeal membrane oxygenation,” “extracorporeal life support,” “cross infection,” “healthcare- associated infection,” “urinary tract infection,” “bloodstream infection,” “catheter-associated infection” and “ventilator-associated pneumonia.” Both controlled vocabulary (e.g., MeSH terms) and free-text keywords were utilized as appropriate for each database, with no language restrictions applied initially. The specific search strategy is provided in Appendix 1.

### 2.3 Quality assessment

Two reviewers systematically trained in evidence-based methods independently assessed the quality of the included literature. Any disagreements were resolved by a third expert reviewer. The methodological quality of clinical guidelines was evaluated using the AGREE II instrument (15), while expert consensus documents were appraised according to the criteria developed by the JBI Center for Evidence-Based Healthcare (2017 edition) (16). Clinical decision resources were assessed using a standardized tool for clinical applicability, and systematic reviews were evaluated with the AMSTAR tool (17). Cohort studies were appraised using the Newcastle-Ottawa Scale (18). Data were extracted by two independent reviewers, and the methodological quality of each included study was graded according to the 2011 Oxford Centre for Evidence-Based Medicine levels of evidence (19).

### 2.4 Data extraction

To ensure the accuracy and consistency of the data extraction process, two reviewers independently extracted data from the included studies. Any discrepancies or disagreements between the reviewers were resolved through consultation with a third reviewer for a final judgment. In cases where conflicting evidence was identified, priority was given to evidence from higher-quality and more recently published sources.

## 3 Results

### 3.1 Literature search results

**Figure 1:**
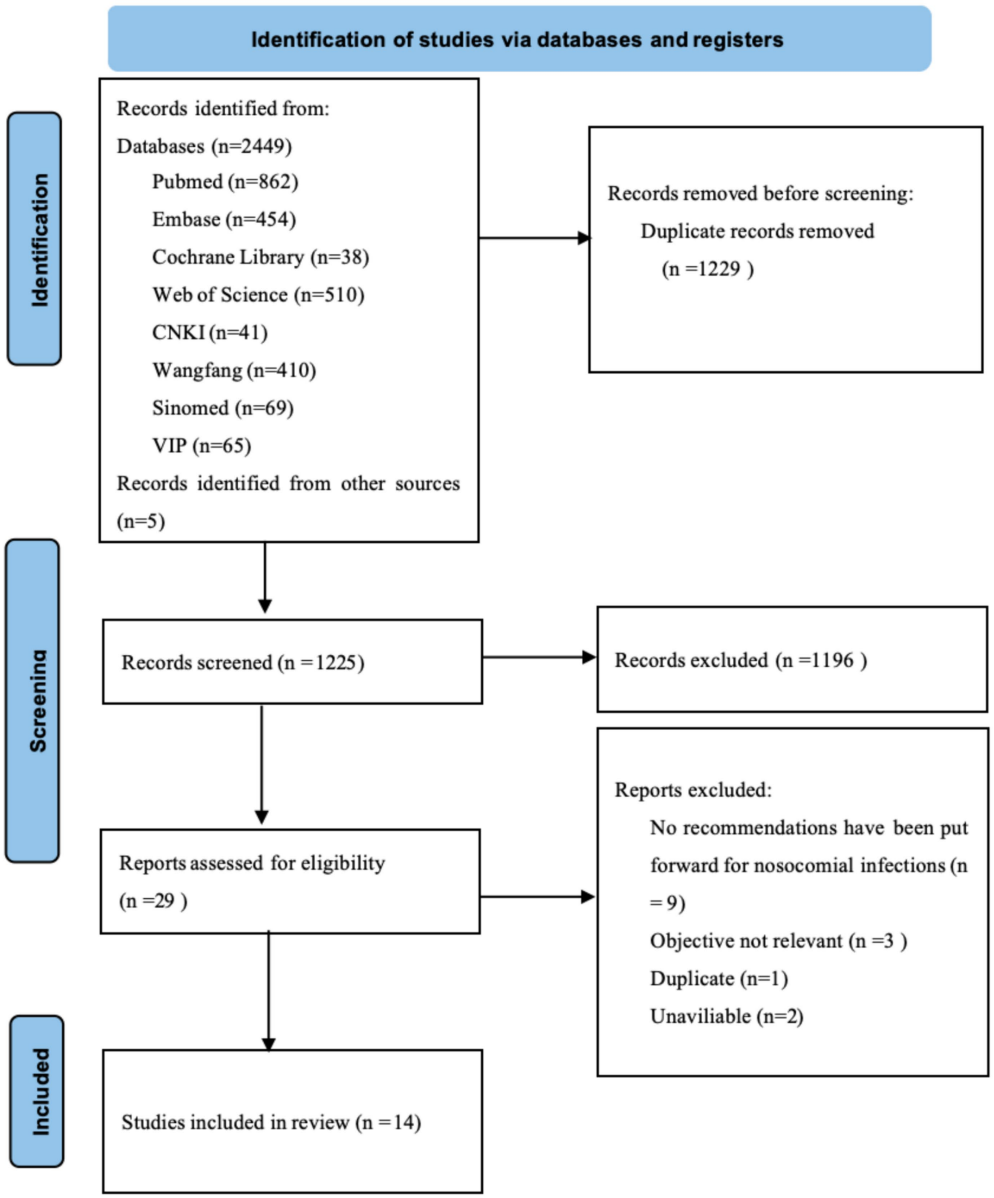
Flow diagram of studies included and excluded at each stage of review.

The study selection process followed the PRISMA guidelines and is summarized in Figure 1. Initially, 2,449 records were identified through database searching, with an additional 5 records retrieved from guideline websites. After removing 1,229 duplicate records using EndNote software, 1,225 publications underwent title and abstract screening. Following this screening, 1,196 records were excluded for not meeting the inclusion criteria. The remaining 29 full-text articles were further assessed for eligibility, of which 15 were excluded. Ultimately, 14 studies met the inclusion criteria, comprising 2 clinical decision resources (20, 21), 4 clinical guidelines (22–25), 4 expert consensus documents (26–29), 2 systematic reviews (4, 30), and 2 cohort studies (31). The characteristics of the included studies are summarized in Table 1.

**Table 1:** Characteristics of included studies (*N* = 14).

Author	Year of publication	Title	Study type	Source
Darryl Abrams (20)	2025	Extracorporeal life support in adults: Management of venovenous extracorporeal membrane oxygenation (V-V ECMO)	Clinical decision	UptoDate
Zhang (26)	2025	Consensus on nursing care for adult extracorporeal membrane oxygenation circulatory assistance	Consensus	CNKI
Taylor M (11)	2025	Use of standardized nasal and skin decolonization to reduce rates of bacteremia in patients undergoing extracorporeal membrane oxygenation	Cohort Study	Pubmed
Klompas M (21)	2024	Treatment of hospital- acquired and ventilator- associated pneumonia in adults	Clinical decision	UptoDate
Extracorporeal Life Support Branch of Chinese Society of Cardiothoracic Vascular Anesthesia (27)	2024	Expert consensus on the prevention and management of infection during extracorporeal membrane oxygenation in adult patients	Consensus	CNKI
National Health Commission of the People’s Republic of China Medical Administration (23)	2024	Technical Operation Standards for Adult Extracorporeal Membrane Oxygenation (2024 Edition)	Guideline	National Health Commission of the People’s Republic of China Medical Administration
Ali Ait Hssain (4)	2024	Incidence, risk factors and outcomes of nosocomial infection in adult patients supported by extracorporeal membrane oxygenation: a systematic review and meta-analysis	Systematic review	Pubmed
Orso D (30)	2024	Do patients receiving extracorporeal membrane-oxygenation need antibiotic prophylaxis? A systematic review and meta-analysis on 7,996 patients	Systematic review	Pubmed
Chinese emergency ECMO research collaboration group (28)	2023	Chinese consensus of emergency experts on percutaneous cannulation for adult extracorporeal cardiopulmonary resuscitation	Consensus	CNKI
Massart N (31)	2023	Multiple-site decontamination to prevent acquired infection in patients with veno-venous ECMO support	Cohort Study	Pubmed
Graeme MacLaren (24)	2022	Extracorporeal Life Support: The ELSO Red Book 6th Edition	Guideline	ELSO
Assmann A (22)	2022	Use of extracorporeal circulation (ECLS/ECMO) for cardiac and circulatory failure –A clinical practice Guideline Level 3	Guideline	Pubmed
Chinese Medical Doctor Association Committee of Extracorporeal Life Support (29)	2018	Consensus on extracorporeal membrane oxygenation for circulatory support in adults	Consensus	CNKI
Extracorporeal Life Support Organization (25)	2017	Extracorporeal Life Support Organization (ELSO) General Guidelines for all ECLS Cases	Guideline	ELSO

CNKI, China National Knowledge Infrastructure; ECLS, Extracorporeal Life Support; ELSO, Extracorporeal Life Support Organization.

### 3.2 Quality assessment of included studies

The methodological quality of the 4 included guidelines (22–25) was appraised, with detailed results presented in Table 2. The 4 expert consensus documents (26–29) were also quality assessed, and the results are shown in Table 3. Both systematic reviews (4, 30) met all quality criteria across assessment domains and were therefore included. The two included cohort studies (11, 31) received a quality score of 8 points. Both clinical decision resources (20, 21) were deemed suitable for inclusion.

**Table 2:** Quality assessment results of guidelines (*N* = 4).

Inclusion in the literature	Standardized score for each field (%)						Number of fields with ≥60% (one)	≥30% of the number of areas (one)	Recommendation level
	Scope purpose	Participants	Rigour	Clarity	Applicability	Independence			
National Health Commission of the People’s Republic of China Medical Administration (23)	97.22%	38.89%	53.13%	94.44%	43.75%	100%	3	6	B
Graeme MacLaren (24)	97.22%	72.22%	71.88%	100%	62.5%	100%	6	6	A
Assmann, A. (22)	88.89%	72.22%	90.63%	100%	60.42%	100%	6	6	A
Extracorporeal Life Support Organization (25)	83.33%	55.56%	67.71%	91.67%	35.42%	89.29%	4	6	B

**Table 3:** Quality assessment results of included consensus (*N* = 4).

Included consensus	①	②	③	④	⑤	⑥	Included
Zhang (26)	Y	Y	Y	Y	Y	Y	Y
Extracorporeal Life Support Branch of Chinese Society of Cardiothoracic Vascular Anesthesia (27)	Y	Y	Y	Y	Y	Y	Y
Chinese emergency ECMO research collaboration group (28)	Y	Y	Y	Y	Y	Y	Y
Chinese Medical Doctor Association Committee of Extracorporeal Life Support (29)	Y	Y	Y	Y	Y	Y	Y

① Are the sources of the ideas clearly marked? (YES/NO). ② Does the opinion come from an influential expert in the field? (YES/NO). ③ Whether the point of view presented is centered on the relevant population interests of the study? (YES/NO). ④ Are the stated conclusions based on the analysis? Are ideas expressed logically? (YES/NO). ⑤ Was there any reference to other literature? (YES/NO). ⑥ Are there any inconsistencies between the ideas presented and the previous literature? (YES/NO). Letter Y, indicates whether criteria ①–⑥ are met and decides whether to include evidence.

### 3.3 Evidence summary

Following literature screening and quality assessment, evidence related to infection prevention and management in ECMO patients was extracted from the 14 included studies. The extracted evidence was initially categorized into three phases: (1) infection prevention during ECMO initiation, (2) infection prevention during ECMO run, and (3) monitoring and treatment of ECMO-related infections. Two reviewers independently performed evidence extraction and categorization. Disagreements were resolved through discussion; if consensus could not be reached, a third reviewer was consulted. Subsequently, the research team conducted iterative discussions to synthesize the evidence by merging similar statements, removing duplicates, and reconciling conflicting recommendations. Through this consensus-building process, the evidence was consolidated into 27 distinct evidence statements. Any persistent disagreements during the synthesis and grading process were resolved by team discussion or consultation with an external expert when necessary. The final 27 evidence statements are presented in Table 4.

**Table 4:** Summary of evidence items.

Category	Subcategory	Evidence items	Evidence level	Grade of recommendation
Infection prevention during ECMO initiation	Risk factor identification	Multiple risk factors for nosocomial infections in ECMO patients have been identified. These include patient-related factors (advanced age, body mass index, underlying comorbidities, higher SOFA and SAPS scores); ECMO-related factors (VV vs. VA mode, duration of ECMO support, ECMO catheter colonization, duration of arterial catheterization); treatment-related factors (duration of mechanical ventilation, hospital length of stay, CPR duration <5 min, hemodialysis); organ dysfunction (acute renal failure, acute hepatic failure); and mechanical complications (4).	3	A
	Procedural preparation and cannulation	A comprehensive sterile barrier should be strictly adhered to during ECMO cannulation, following a thorough assessment of the procedural environment (24, 27).	1	A
		Ultrasound-guided percutaneous cannulation is recommended as the preferred approach for ECMO initiation (24, 27, 28).	1	A
		The use of a mechanical chest compression device is advised during extracorporeal cardiopulmonary resuscitation (eCPR) while establishing ECMO flow (27).	5	B
		The use of razors for preoperative hair removal is not recommended. Electric clippers should be used for surgical site hair management if necessary (24, 27).	5	B
	Environmental and staffing management	Implementing single-room isolation and assigning dedicated nursing staff for ECMO patients is recommended whenever possible (22, 26, 27).	5	B
Infection prevention during ECMO run	Skin and mucosal decontamination	For oral care in ECMO patients, gentle and thorough non-pharmacological cleaning is preferred over the routine use of chlorhexidine to protect the oral mucosa (24, 27).	3	B
		Chlorhexidine should be used for disinfecting the ECMO circuit and cannula insertion sites in patients receiving ECMO support (24, 27).	4	B
		ECMO patients should receive daily bathing with chlorhexidine wipes combined with once-daily nasal application of mupirocin (21, 24, 31).	4	B
	Respiratory management	Flexible bronchoscopy should be utilized for pulmonary secretion clearance and specimen collection for culture in ECMO patients (24).	4	B
	Cannula site management and skin protection	Use transparent dressings on ECMO cannulation sites for visual monitoring. Daily assess catheter and dressing/suture integrity. All changes/reinforcements must be aseptic per protocol (20, 24, 27).	5	A
		Hydrocolloid or foam dressings should be placed beneath ECMO cannulas to prevent medical device-related pressure injuries (23, 28).	5	A
	Circuit integrity and invasive device management	Minimize circuit breaches; use needleless connectors when access is required (24, 27).	5	A
		Non-essential central venous catheters and other invasive devices should be removed as early as possible during ECMO support (24, 27).	5	A
	Patient positioning and early rehabilitation	Ensure adequate staffing and coordinated assistance during patient positioning to prevent ECMO cannula dislodgement (24).	5	A
		Early mobilization and chest physiotherapy should be initiated promptly in ECMO patients when clinically feasible (24).	5	B
		Enteral nutrition should be established early, preferably via gastric or post-pyloric route within 48 h of ECMO initiation (27).	4	B
	Sedation and general infection prevention bundles	A light sedation strategy should be implemented as soon as the patient’s condition is stabilized during ECMO support (25, 27).	4	A
		Adhere to locally adapted care bundles for infection prevention in critically ill patients, including CLABSI, CAUTI, VAP, and SSI (24, 26, 27).	3	A
Monitoring and treatment of ECMO-related infections	Infection surveillance and monitoring	Closely monitor ECMO patients for signs of sepsis and conduct prompt evaluation when indicated (24, 27).	2	A
		Maintain vigilant infection surveillance during ECMO. When infection is suspected, obtain repeat broad-spectrum cultures. Blood cultures should be indication-based, not routine daily; other site cultures (e.g., respiratory, urinary, catheter) may be increased as clinically indicated (24, 27).	3	B
		ECMO temperature >37.5 °C suggests infection. Monitor biomarkers (PCT, CRP, IL-6, IL-10) for early warning (23).	5	A
	Antimicrobial prophylaxis and empirical therapy	Routine prophylactic antibiotics are not recommended during ECMO initiation. For postoperative patients, administer surgical prophylaxis per procedure type and institutional guidelines (24, 27, 29, 30).	3	B
		Initiate empirical broad-spectrum antibiotics immediately after obtaining cultures from suspected infection sites. Tailor the regimen to local epidemiology and antibiogram data; consider adjunctive antifungal therapy when indicated (24).	4	B
	Management of cannula-related infections	Treat ECMO cannula-related infections with systemic antibiotics. Do not routinely remove or exchange the cannula. Only remove as a last resort for source control in refractory septic shock (20).	5	B
	Antibiotic pharmacokinetics and therapeutic drug monitoring	The ECMO circuit alters antibiotic pharmacokinetics, especially for lipophilic, highly protein-bound drugs with large volume of distribution, often causing subtherapeutic levels. Therapeutic drug monitoring is recommended, and consultation with a clinical pharmacist is advised in complex cases (24, 29).	5	A
	Weaning and circuit removal	Perform daily assessments for the feasibility of discontinuing ECMO support. The circuit should be removed as early as possible once the patient meets weaning criteria (27).	5	A

ECMO, extracorporeal membrane oxygenation; CLABSI, central line-associated bloodstream infections; CAUTI, catheter-associated urinary tract infections; VAP, ventilator-associated pneumonia; and SSI, surgical site infections.

## 4 Discussion

Based on a systematic review of existing evidence, this study synthesized 27 evidence statements, forming a comprehensive strategy for infection prevention and management in ECMO patients. Although most evidence was graded at levels 3 to 5, the lower grading does not diminish the importance of the recommendations. These statements represent consensus developed through clinical experience and pathophysiological rationale, remaining significant for guiding clinical practice and ensuring patient safety.

The critical condition of ECMO patients makes the identification of specific risk factors for NI essential for developing targeted prevention and control strategies. A recent systematic review identified prolonged mechanical ventilation, underlying comorbidities, BMI, advanced age, extended ECMO duration, and ECMO mode as significant risk factors for NI in this population (4). However, the related evidence primarily stems from observational studies, with most evidence levels ranging from moderate to low. This is mainly due to the methodological challenges faced when researching such critically ill populations: high patient heterogeneity, complex treatment combinations, and the difficulty of implementing strict randomized controlled trial designs, which inherently hinder the establishment of clear causal relationships. Juthani et al. (32) further specified that ECMO support exceeding 10 days represents a primary risk factor for healthcare-associated infections. Prolonged ECMO support not only increases exposure to potential infections but also extends the overall disease course. While different ECMO modalities may influence infection rates (24), it must be emphasized that ECMO support itself, regardless of its specific configuration, constitutes a significant risk factor for NI. This risk primarily stems from the patients’ underlying critical illness and the intensity of invasive support required. Currently, there is a notable absence of widely validated and universally adopted predictive models for ECMO-related infections. Most published models remain single-center, retrospective investigations. Therefore, future efforts should focus on systematically collecting high-quality data through prospective, multicenter cohort studies and integrating novel biomarkers and real-time monitoring indicators to overcome the limitations of current research. With advancing machine learning algorithms, researchers now have the opportunity to develop robust infection risk prediction models for ECMO patients through multivariate analysis of comprehensive clinical datasets.

Preventing NI associated with ECMO is crucial, yet there remains a lack of standardized guidelines specifically targeting infection prevention during ECMO support. A retrospective study demonstrated that even when the same bundle of preventive measures was implemented, the infection rate among ECMO patients remained higher than that of general intensive care unit (ICU) patients (13). This finding underscores the necessity of developing infection control strategies specifically tailored for ECMO patients, beyond conventional care protocols. The principle of aseptic technique must be maintained throughout the entire ECMO management process. Prior to ECMO initiation, a standardized sterile operating procedure should be strictly followed. For patients requiring ongoing resuscitation, such as those undergoing extracorporeal cardiopulmonary resuscitation, the use of a mechanical chest compression device is recommended (24, 27). This approach not only ensures high-quality circulatory support but also maintains a stable operative environment, thereby avoiding contamination of the sterile field that may occur during manual chest compressions.

The ECMO circuit, as a long-term indwelling high-risk vascular access device, requires a multi-dimensional defense strategy for systematic infection prevention and control. It is important to note that there is limited high-level evidence supporting the specific measures mentioned above (such as particular dressing types or extubation timeframes), and many recommendations are based on clinical experience and observational data. This reflects the significant challenges in conducting prospective randomized controlled studies to evaluate the independent effectiveness of single infection prevention and control measures within the complex intervention that is ECMO. Cannulation itself compromises the integrity of the skin barrier, and the risk of infection correlates directly with the duration of catheterization (2). The application of transparent semipermeable film dressings over puncture sites provides an effective microbial barrier while enabling continuous visual assessment for early detection of infection signs. ELSO guidelines recommend avoiding unnecessary circuit access to maintain system integrity. All essential connections should utilize needleless connectors, with interfaces securely sealed using aseptic techniques to effectively block microbial invasion pathways (24, 33). Manerikar et al. (34) demonstrated that central venous catheter indwelling exceeding 8 days in ECMO patients was associated with higher rates of bloodstream infection and oxygenator secondary colonization. Therefore, daily assessment and prompt removal of non-essential lines are crucial for reducing potential infection portals at the source. Comprehensive skin management remains equally indispensable. Prophylactic dressings (such as hydrocolloid or foam dressings) should be placed beneath ECMO cannulas and monitoring devices to maintain clean, dry skin. This approach not only prevents pressure injuries but also serves as a critical measure for preserving the body’s natural barrier function.

In refined skin and mucosal decolonization strategies, chlorhexidine serves as a critical broad-spectrum antiseptic agent. Systematic reviews indicate that chlorhexidine bathing can reduce the incidence of bloodstream infections by approximately 29% (IRR = 0.71, 95% CI 0.51–0.9) (35). A cluster-randomized trial published in JAMA demonstrated that combining chlorhexidine bathing with nasal mupirocin decolonization effectively reduced clinical culture positivity for *staphylococcus aureus* and methicillin-resistant *staphylococcus aureus* among ICU patients (36). However, evidence applicability to ECMO patients remains unclear, and existing results are conflicting. One study found that chlorhexidine plus mupirocin failed to reduce bloodstream infections in ECMO patients but unexpectedly increased enterococcal infections (11). Conversely, another study reported that a multi-site decontamination regimen including topical antibiotics significantly reduced infections (31). These discrepancies may stem from differences in intervention intensity and study populations. An uncontrolled before-after study suggested that wiping exposed ECMO circuits with 2% chlorhexidine gluconate was associated with reduced rates of catheter-related bloodstream infections and microbial colonization (37).

It is important to note that high-quality evidence specifically for ECMO patients is particularly lacking in these studies. This is partly due to the complex and highly heterogeneous clinical status of this population, which makes it difficult to conduct rigorous randomized controlled trials. Additionally, in clinical practice, chlorhexidine is often used as part of a “bundle strategy” for infection prevention and control, making its isolated effect challenging to precisely evaluate. Therefore, given the methodological limitations of this study, large-scale, multicenter, randomized controlled trials are needed to validate these findings. The application of chlorhexidine requires careful benefit–risk consideration, particularly at vulnerable mucosal sites. Unlike skin antisepsis, chlorhexidine should not be routinely recommended as the first-line agent for oral care. Studies have shown that chlorhexidine mouthwash may disrupt the oral microbiome balance and cause mucosal irritation (38). Compared to pharmacological interventions, gentle yet thorough physical cleaning using a soft-bristled toothbrush combined with suction appears safer and more effective for maintaining oral hygiene in this patient population. Therefore, for the oral care of ECMO patients, the approach should be based on gentle physical cleaning supplemented by pharmacological interventions when necessary, aiming to achieve effective infection prevention and control while maximizing the maintenance of oral microbiome balance and mucosal integrity.

Antimicrobial stewardship remains a central yet challenging aspect of NI prevention and management in ECMO patients. Antimicrobial stewardship is defined as a set of coordinated interventions designed to improve and measure the appropriate use of antimicrobial agents by promoting the optimal selection of drug regimens, including dosage, duration of therapy, and route of administration (39). It is noteworthy that many key recommendations in this field are based on evidence of moderate to low quality. This stems primarily from methodological challenges: ECMO support itself constitutes a highly complex life-sustaining intervention, making it extremely difficult to independently assess the net benefit of antibiotic prophylaxis strategies or specific dosing regimens; simultaneously, the critical condition and heterogeneity of the patient population limit the feasibility of conducting large-scale prospective randomized controlled trials. While ‌sepsis frequently complicates ECMO therapy, consensus is still lacking regarding whether antibiotic prophylaxis reduces mortality or infection incidence in this population (24). While ELSO guidelines do not recommend routine prophylactic antibiotics for ECMO patients, about half of U. S. centers (40) and 39% of Japanese hospitals (41) continue to implement prophylactic antibiotic use in practice. A recent systematic review demonstrated that antibiotic prophylaxis had no significant effect on 30-day mortality in ECMO patients (very low certainty evidence) but was associated with a reduced risk NI (30). Given that all included studies were retrospective with limited evidence quality, high-quality prospective studies are urgently needed to further validate these findings.

Notably, emerging evidence indicates significant differences in the microbial spectrum and resistance severity of pathogens infecting ECMO patients compared to general ICU populations (42). Multidrug-resistant *Acinetobacter baumannii* and *Klebsiella pneumoniae* demonstrate higher prevalence and greater resistance intensity. Consequently, clinicians managing suspected infections in ECMO patients should prioritize coverage against these more resistant pathogens, adopting more aggressive and targeted empirical antibiotic strategies. The ECMO circuit itself significantly alters pharmacokinetic parameters through drug adsorption (particularly lipophilic agents) to its extensive surface area, resulting in increased volume of distribution and reduced clearance (43). These alterations complicate accurate drug dosing, making therapeutic drug monitoring essential during ECMO support. Therapeutic drug monitoring is the clinical practice of measuring drug levels in the bloodstream to optimize individual dosing (44). While reliable dosing guidelines for critically ill adults on ECMO remain limited, recent research has focused on drug pharmacokinetics and serum concentrations in this population (45). Integrating clinical pharmacists into the core team and advancing therapeutic drug monitoring implementation provide crucial support for dose optimization. Furthermore, novel software systems are being developed (46, 47) that integrate patient-specific data with real-time drug concentrations to create intelligent clinical decision support systems, offering practical solutions for precision medication management in ECMO patients.

ECMO infection prevention requires context-specific implementation: resource-replete hospitals should implement comprehensive bundle measures (checklists, dedicated team, monitoring, simulation training); resource-limited hospitals should prioritize core measures (hand hygiene, sterile barriers, chlorhexidine disinfection, line assessment), use simplified checklists, and collaborate with regional centers. Phased implementation and regular quality review are fundamental.

## 5 Strengths and limitations

This study systematically synthesized the best available evidence across three key domains, namely prevention, control, and treatment of NI in ECMO patients, to develop a comprehensive management strategy with high clinical applicability. Its primary strength lies in its clinical relevance and coverage of the entire patient management pathway. By consolidating fragmented evidence into clear recommendations with explicit grading of evidence levels and recommendation strengths, it provides healthcare professionals with an intuitive and actionable framework for practice.

This study has several limitations. First, the varying definitions of ECMO-associated nosocomial infections across studies, coupled with geographical differences in the causative pathogens and variations in patient population characteristics, pose challenges to establishing a unified global standard. Second, the current evidence base remains relatively weak, with most recommendations relying on observational studies, single-center experiences, or expert consensus. The lack of support from high-quality randomized controlled trials limits the reliability of some recommendations. Furthermore, the external validity and generalizability of the findings require further validation. Whether the summarized strategies are fully applicable to medical centers with varying levels of resources and expertise still needs to be tested through localized implementation and outcome assessment. Future research should focus on conducting high-quality multicenter clinical studies and exploring the translation of such evidence into quantifiable clinical pathways and intelligent decision-support systems. These efforts will be crucial for continuously optimizing infection management in ECMO patients.

## 6 Conclusion

This study consolidates current best evidence to establish a comprehensive management framework for infection prevention, monitoring, and treatment in ECMO patients. While many recommendations are constrained by limited evidence grades, the core value lies in synthesizing fragmented clinical research and practical consensus into a structured action guide, providing crucial reference for clinical practice. The evidence indicates that successful infection control relies on an interlinked systemic strategy: from strict aseptic techniques and barrier protection before cannulation, through active surveillance and refined care of skin, mucous membranes, and catheter sites during support, to precise anti-infective therapy based on pharmacokinetic principles. Future work should focus on well-designed multicenter studies to address evidence gaps in key interventions. Only through continuous cycles of “evidence guiding practice and practice informing evidence” can we achieve sustained improvement in infection control outcomes and overall care quality for ECMO patients.
