# Supplementary material for: Prevention and management of nosocomial infections in patients undergoing extracorporeal membrane oxygenation: a summary of best evidence
Source: Front Med (Lausanne). 2026 May 15;13:1819134. doi: 10.3389/fmed.2026.1819134 (PMC13219359; doi:10.3389/fmed.2026.1819134)
Supplement: Supplementary file 1 [file Table_1.DOCX]

**Pubmed：862 results**

(("Extracorporeal Membrane Oxygenation" [MeSH] OR "oxygenators, membrane"[Title/Abstract] OR "ECMO" [Title/Abstract] OR "extracorporeal life support" [Title/Abstract] OR "ECLS" [Title/Abstract] OR "membrane oxygenator*" [Title/Abstract] OR "Life Support, Extracorporeal ECMO Treatment*" [Title/Abstract]) AND ("Bacteremia" [MeSH] OR "Catheter-Related Infections" [MeSH] OR "Gram-Positive Bacterial Infections" [Title/Abstract] OR "pneumonia, ventilator associated" [MeSH] OR "ventilator-associated pneumonia" [Title/Abstract] OR "Septic" [MeSH] OR "Shock, Septic" [Title/Abstract] OR "septic shock" [Title/Abstract] OR "Urinary Tract Infections" [MeSH] OR "urinary tract infection*" [Title/Abstract] OR "bloodstream infection*" [Title/Abstract] OR "catheter related infection*" [Title/Abstract] OR "catheter associated infection*" [Title/Abstract] OR "device associated infection*" [Title/Abstract] OR "fungal infection*" [Title/Abstract] OR "surgical wound infection" [MeSH] OR "surgical site infection" [Title/Abstract] OR "nosocomial infection*" [MeSH] OR "Cross Infection" [MeSH] OR "healthcare associated infection*" [Title/Abstract] OR "hospital infection*" [Title/Abstract] OR "fungemia*" [Title/Abstract] ))

**EMBAS: 454 results**

('extracorporeal membrane oxygenation':ti,ab OR 'extracorporeal life support':ti,ab OR 'ecmo':ti,ab OR 'ecls':ti,ab) AND ('healthcare associated infection':ti,ab OR 'nosocomial infection':ti,ab OR 'cross infection':ti,ab OR 'ventilator-associated pneumonia':ti,ab OR 'urinary tract infection':ti,ab OR 'bloodstream infection':ti,ab)

**Cochrane Library: 38 results**

(extracorporeal membrane oxygenation):ti,ab,kw OR (extracorporeal life support):ti,ab,kw OR (ECMO):ti,ab,kw OR (ECLS):ti,ab,kw AND (healthcare associated infection):ti,ab,kw OR (nosocomial infection):ti,ab,kw OR (cross infection):ti,ab,kw OR (ventilator-associated pneumonia):ti,ab,kw OR (urinary tract infection):ti,ab,kw OR (bloodstream infection):ti,ab,kw

**Web of science: 510 results**

#1 (((TS=(extracorporeal membrane oxygenation)) OR TS=(extracorporeal life support )) OR TS=(ECMO )) OR TS=(ECLS)

#2 (((((TS=(healthcare associated infection)) OR TS=(nosocomial infection)) OR TS=(cross infection)) OR TS=(ventilator-associated pneumonia)) OR TS=( urinary tract infection)) OR TS=(bloodstream infection)

#3 #1 AND #2

**CNKI: 41 results**

SU = ('Extracorporeal Membrane Oxygenation (ECMO)' + 'Extracorporeal Life Support') AND SU = ('Nosocomial Infection' + 'Hospital Infection' + 'Bloodstream Infection' + 'Urinary Tract Infection' + 'Ventilator-Associated Pneumonia')

**Wangfang: 410 results**

(Title:("Extracorporeal Life Support" OR "Extracorporeal Membrane Oxygenation" OR "ECMO") OR Keywords:("Extracorporeal Life Support" OR "Extracorporeal Membrane Oxygenation" OR "ECMO")) AND (Title:("Nosocomial Infection" OR "Hospital Infection" OR "Bloodstream Infection" OR "Urinary Tract Infection" OR "Ventilator-Associated Pneumonia" OR "Catheter-Related Infection" OR "Infection") OR Keywords:("Nosocomial Infection" OR "Hospital Infection" OR "Bloodstream Infection" OR "Urinary Tract Infection" OR "Ventilator-Associated Pneumonia" OR "Catheter-Related Infection" OR "Infection"))

**VIP: 65 results**

(T=('Extracorporeal Membrane Oxygenation' OR 'ECMO' OR 'Extracorporeal Life Support') OR K=('Extracorporeal Membrane Oxygenation' OR 'ECMO' OR 'Extracorporeal Life Support') OR R=('Extracorporeal Membrane Oxygenation' OR 'ECMO' OR 'Extracorporeal Life Support')) AND (T=('Nosocomial Infection' OR 'Hospital Infection' OR 'Bloodstream Infection' OR 'Catheter-Related Bloodstream Infection' OR 'Urinary Tract Infection' OR 'Ventilator-Associated Pneumonia' OR 'Cannulation Site Infection' OR 'Respiratory Tract Infection') OR K=('Nosocomial Infection' OR 'Hospital Infection' OR 'Bloodstream Infection' OR 'Catheter-Related Bloodstream Infection' OR 'Urinary Tract Infection' OR 'Ventilator-Associated Pneumonia' OR 'Cannulation Site Infection' OR 'Respiratory Tract Infection') OR R=('Nosocomial Infection' OR 'Hospital Infection'))

**Sinomed: 69 results**

(("Extracorporeal Membrane Oxygenation"[MeSH Terms] OR "Extracorporeal Membrane Oxygenation"[Common Fields] OR "Extracorporeal Membrane Oxygenation"[Common Fields] OR "Extracorporeal Life Support"[Common Fields] OR "ECMO"[Common Fields]) AND ("Cross Infection"[MeSH Terms] OR "Cross Infection"[Common Fields] OR "Nosocomial Infection"[Common Fields] OR "Hospital Infection"[Common Fields] OR "Iatrogenic Infection"[Common Fields])) OR (("Extracorporeal Membrane Oxygenation"[MeSH Terms] OR "Extracorporeal Membrane Oxygenation"[Common Fields] OR "Extracorporeal Membrane Oxygenation"[Common Fields] OR "Extracorporeal Life Support"[Common Fields] OR "ECMO"[Common Fields]) AND ("Bacteremia"[MeSH Terms] OR "Sepsis"[MeSH Terms] OR "Urinary Tract Infections"[MeSH Terms] OR "Ventilator-Associated Pneumonia"[MeSH Terms] OR "Bloodstream Infection"[Common Fields] OR "Urinary Tract Infection"[Common Fields] OR "Ventilator-Associated Pneumonia"[Common Fields]))
